# Supplementary material for: Smartphone-Controlled Aptasensor for Voltammetric Detection of Patulin in Apple Juice
Source: Sensors (Basel). 2024 Jan 24;24(3):754. doi: 10.3390/s24030754 (PMC10856927; doi:10.3390/s24030754)
Supplement: Supplementary file 1 [file sensors-24-00754-s001.zip › sensors-2792389-supplementary.pdf]

Supplementary Material

Smartphone-Controlled Aptasensor for Voltammetric Detection of Patulin in Apple Juice

Arzum Erdem* and Huseyin Senturk

Analytical Chemistry Department, Faculty of Pharmacy, Ege University, Bornova,
35100, Izmir, TURKEY

***Corresponding author (A. Erdem):** arzum.erdem@ege.edu.tr

Safety information

Patulin: GSH06; Hazard Classifications: Acute Tox. 2 Oral - Skin Irrit. 2; Hazard Statements: H300 - H315; Storage Class Code: 6.1A - Combustible, acute toxic Cat. 1 and 2 / very toxic hazardous materials; WGK: WGK3

Fumonisin B1: GHS02,GHS07; Hazard Classifications: Acute Tox. 4 Oral - Eye Irrit. 2 - Flam. Liq. 2; Hazard Statements: H225 - H302 - H319; Storage Class Code: 3 - Flammable liquids; WGK: WGK 2

Deoxynivalenol: GHS02,GHS07; Hazard Classifications: Acute Tox. 4 Dermal - Acute Tox. 4 Inhalation - Acute Tox. 4 Oral - Eye Irrit. 2 - Flam. Liq. 2; Hazard Statements: H225 - H302 + H312 + H332 - H319; Storage Class Code: 3 - Flammable liquids; WGK: WGK 2

Ochratoxin A: GHS02,GHS07; Hazard Classifications: Acute Tox. 4 Dermal - Acute Tox. 4 Inhalation - Acute Tox. 4 Oral - Eye Irrit. 2 - Flam. Liq. 2; Hazard Statements: H225 - H302 + H312 + H332 - H319; Storage Class Code: 3 - Flammable liquids; WGK: WGK 2

Table S1. The average current values (*n*=3) of redox probe with % RSD and the change ratio (%) at current value obtained before/after aptamer immobilization onto the electrode (PGE) surface at different concentrations of DNA aptamer.

	**I (μA) and RSD (%)**	**Change ratio (%) at current**
PGE (Control group)	212.59 ± 2.75<br>1.29 %	
0.25 μg/mL Aptamer/PGE	203.52 ± 0.60<br>0.29 %	4.27 Decrease
1 μg/mL Aptamer/PGE	194.76 ± 17.29<br>8.88 %	8.39 Decrease
2.5 μg/mL Aptamer/PGE	141.11 ± 6.30<br>4.46 %	33.62 Decrease
5 μg/mL Aptamer/PGE	149.55 ± 13.72<br>9.17 %	29.65 Decrease

Table S2. The average current values (*n*=3) of redox probe with % RSD and the change ratio (%) at current value obtained before/after aptamer immobilization onto the electrode surface in different immobilization times and after interaction of aptamer with PAT.

	I_{APT} (µA) and RSD (%)	$I_{APT\text{-}PAT}$ (µA) and RSD (%)	Change ratio (%) at current
30 min immobilization	142.07 ± 2.10<br>1.48 %	149.41 ± 13.86<br>9.28 %	5.17 Increase
60 min immobilization	141.11 ± 6.30<br>4.46 %	99.62 ± 13.41<br>13.46 %	29.41 Decrease

Table S3. The average current values (*n*=3) of redox probe with % RSD and the change ratio (%) at current value obtained before/after aptamer interaction with PAT in different interaction times.

	I_{APT} (µA) and RSD (%)	$I_{APT\text{-}PAT}$ (µA) and RSD (%)	Change ratio (%) at current
15 min interaction		106.84 ± 21.63<br>20.25 %	24.29 Decrease
30 min interaction	141.11 ± 6.30<br>4.46 %	99.62 ± 13.41<br>13.46 %	29.41 Decrease
60 min interaction		102.73 ± 9.30<br>9.05 %	27.20 Decrease

Limit of detection

In this study, the limit of determination was calculated according to the IUPAC method [1]. The analyte's signal at the detection limit (Sdl) is given by:

$$Sdl = Sreag + k * \sigma reag,$$

where Sreag is the electrochemical signal for a blank, σreag is the known standard deviation for the blank's electrochemical signal (n_σ=3 for buffer medium and n_σ=3 for apple juice medium).

k is a numerical factor chosen according to the confidence level desired. In this study k value was selected as k=3 (99.86 % confidence level) according to the Long and Winefordner [2].

In our study, Sreag is the electrochemical signal for a blank (aptamer control), σreag is the known standard deviation for the electrochemical signal of aptamer control (n_σ=3 for buffer medium and n_σ=3 for apple juice medium). The electrochemical signal of redox probe (current; I, µA) used to calculate LOD were presented in Table S4 and Table S5 for buffer medium and apple juice medium, respectively. The detection limit of aptasensor was achieved as 0.18 pg/mL in buffer medium and 0.47 pg/mL in apple juice medium by the data given in Table S4 and Table S5, formula and calibration curves.

Table S4. The electrochemical signal of redox probe presenting the values (I, µA) of the blank for three times in buffer medium.

Number	I (µA)	Average (µA)	SD (µA)	RSD (%)
1	121.18			
2	120.32	120.31	0.88	0.73
3	119.42			

Table S5. The electrochemical signal of redox probe presenting the values (I, µA) of the blank for three times in apple juice medium.

Number	I (µA)	Average (µA)	SD (µA)	RSD (%)
1	146.54			
2	147.18	146.96	0.37	0.25
3	147.17			

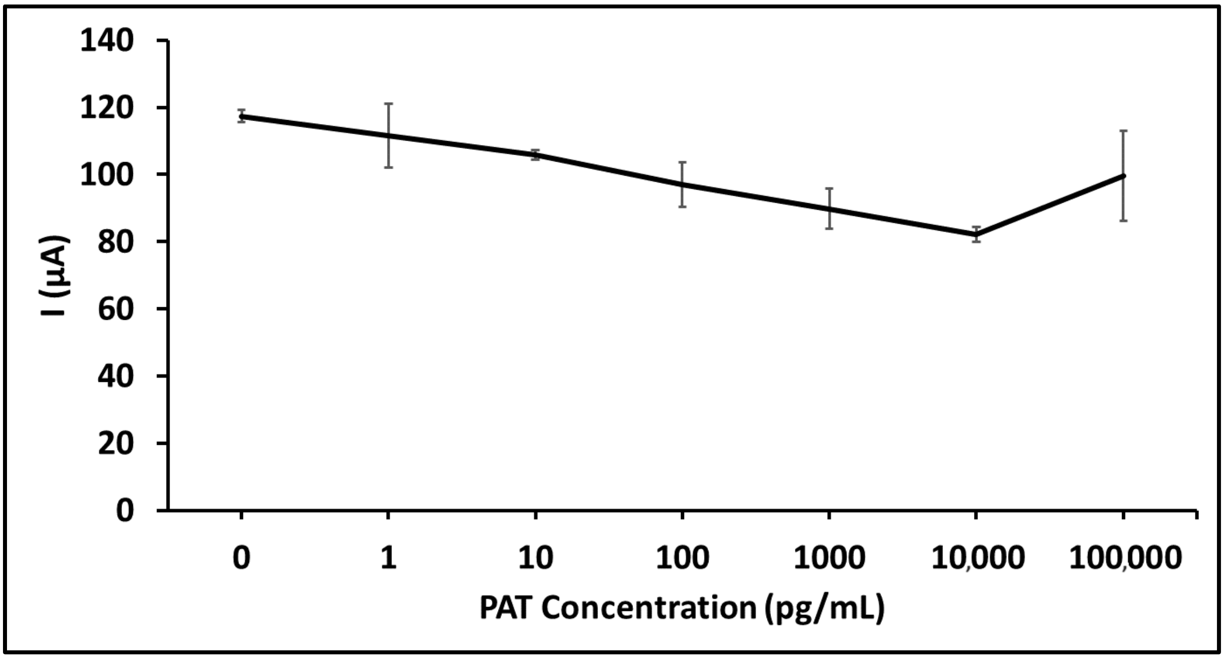

Figure S1. Line graph representing the average current values (*n*=3) of redox probe obtained with increasing concentration of PAT in buffer medium (50 mM PBS, pH 7.4).

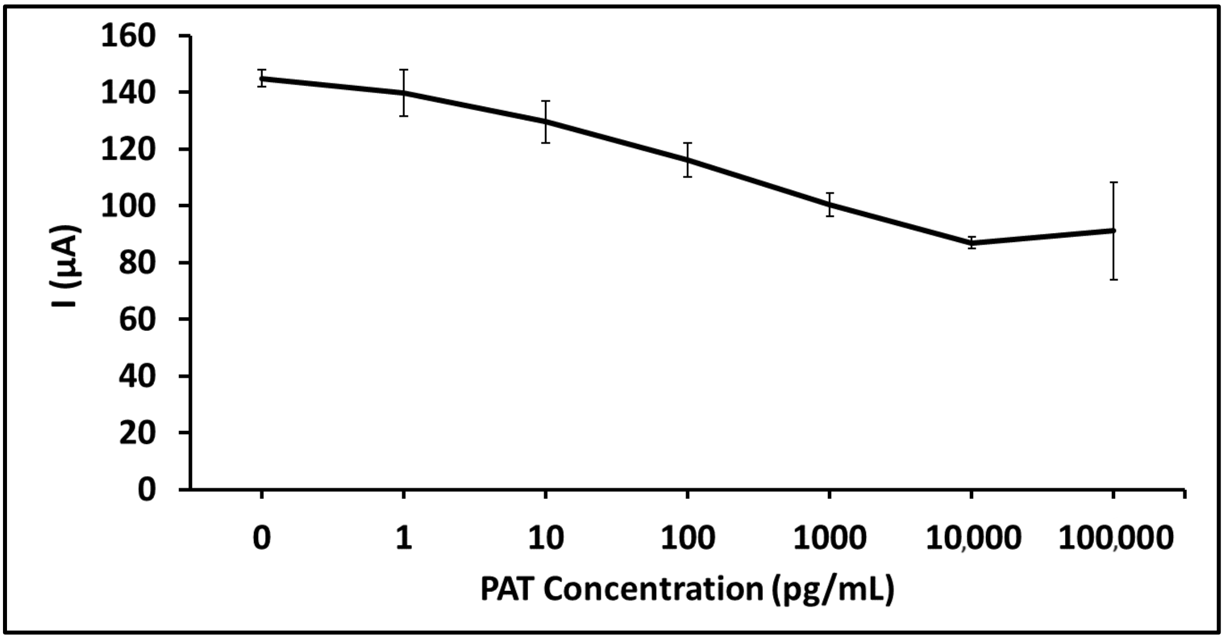

Figure S2. Line graph presenting the average current values (*n*=3) of redox probe measured by smartphone connected to portable device while increasing the concentration of PAT (1 to 10^5 pg/mL) prepared in diluted apple juice medium.

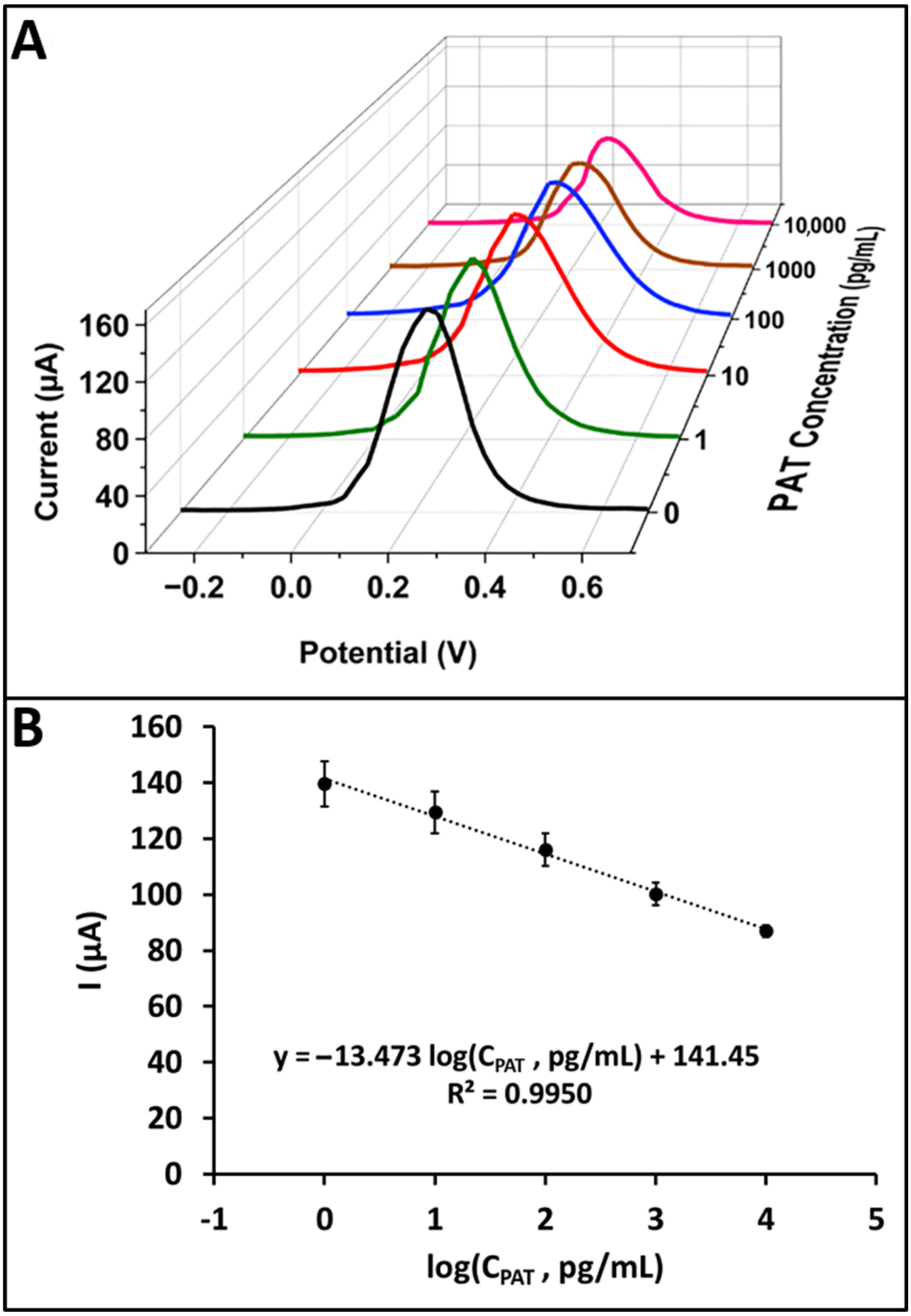

Figure S3. (A) Representative voltammograms of redox probe signal recorded by using a smartphone integrated to portable device in the concentration range of PAT varying from 1 pg/mL to 10^4 pg/mL prepared in diluted apple juice medium (1:50). The voltammograms in black, green, red, blue, brown and pink colour represent 0 pg/mL (blank), 1 pg/mL, 10^1 pg/mL,

10^2 pg/mL, 10^3 pg/mL and 10^4 pg/mL PAT, respectively. (B) Calibration plot based on the data for detection of PAT in apple juice medium by using smartphone integrated to portable device.

Table S6. Reproducibility of PAT aptasensor using a smartphone integrated to portable device in apple juice medium in three different days.

10,000 pg/mL PAT	I (µA)	RSD (%, *n*=6)
1st day	85.81	5.23
	84.61	
2nd day	78.25	
	89.44	
3rd day	78.79	
	85.67	

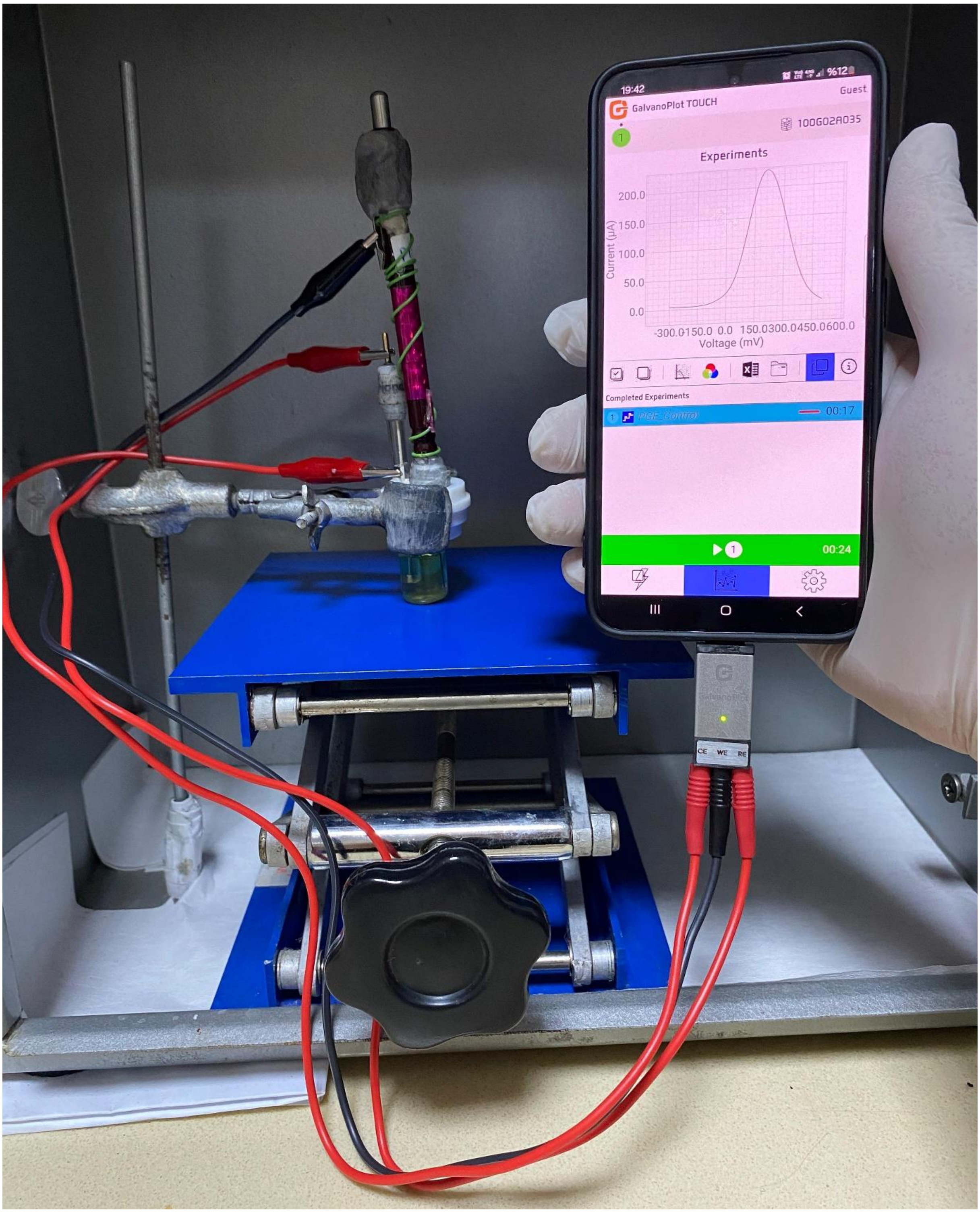

Figure S4. Picture of the set-up of the smartphone integrated portable device.

References

1. IUPAC Nomenclature, Symbols, Units and Their Usage in Spectrochemical Analysis—Iii. Analytical Flame Spectroscopy and Associated Non-Flame Procedures. *Pure Appl. Chem.* **1976**, *45*, 105–123, doi:10.1351/pac197645020105.

2. Long, G.L.; Winefordner, J.D. Limit of Detection A Closer Look at the IUPAC Definition. *Anal. Chem.* **1983**, *55*, 712–714.
